# Synthesis and Characterization of Negative-Tone Photosensitive Polyimides with Low Coefficient of Thermal Expansion for Packaging Applications

**DOI:** 10.3390/polym16131805

**Published:** 2024-06-26

**Authors:** Peng Zhang, Hehe Wang, Pengcheng Xia, Xiaolei Chen, Wei Zhao, Chengqian Wang, Xiao Meng, Bin Jia

**Affiliations:** 1 China Electronics Technology Group Corporation No.58 Research Institute, Wuxi 204035, China; 15961796508@163.com (P.Z.); 13236500356@163.com (H.W.); xpc1011@163.com (P.X.); chenlei-198@126.com (X.C.); zwei370@163.com (W.Z.); 2 Minseoa Advanced Polyimide Corporation, Beijing 101300, China; 18353112790@163.com

**Keywords:** photosensitive polyimide, tripartite structure, low coefficient of thermal expansion, mechanical properties

## Abstract

Negative-tone photosensitive polyimides (PSPIs) with a low coefficient of thermal expansion (CTE) were prepared by dissolving polyimide precursor-poly(amide ester) (PAE) resins, photoinitiators, photocrosslinkers and other additives in organic solvents. Using triamine as a monomer and dianhydride and diamine as polycondensates, tri-branched structure PAE resins with different molecular weights named PAE-1~5 were prepared. A series of corresponding PSPI films named PSPI-1~5 were prepared from PAE-1~5 resins with the same formulation, respectively. The PSPI-1~5 films prepared from resins of this structure have excellent mechanical, thermal and electrical properties after being thermally cured at 350 °C/2 h in nitrogen. The PSPI-1~5 films’ coating solution also show good photolithographic performance and are able to obtain photolithographic patterns with a resolution of about 10 μm after homogenization, exposure and development. Among the PSPI-1~5 films, PSPI-2 has the most excellent lithographic properties with a weight average molecular weight (M_w_) of 2.9 × 10^4^ g/mol, a CTE of 41 ppk/°C, a glass transition temperature (T_g_) of 343 °C and a 5% weight loss temperature (T_d5_) of 520 °C, making it suitable for industrial scale-up. The mechanical properties of elongation at breakage of 42.4%, tensile moduli of 3.4 GPa and tensile strength of 153.7 MPa were also measured.

## 1. Introduction

With the rapid development of the information age, 5G communication technology has entered into people’s daily life, and high-frequency and high-speed communication is one of the major development trends of the future. Polyimide (PI) is widely used in the field of microelectronics and integrated circuits due to its excellent mechanical properties, dielectric properties, chemical stability and heat resistance. Among them, the photosensitive polyimide photoresist products not only have the excellent physical properties of PI, compared with the traditional photoresists, but can also significantly shorten the process, so it can be used as a semiconductor device passivation film, surface protection film, interlayer insulating film, etc., and become an important raw material in the chip packaging industry [1,2,3,4,5]. PSPI as a photopolymer resin composition is mainly composed of base resin (including soluble polyimide and precursors such as poly(amic acid) (PAA) and PAE), a solvent and a photoinitiator. Commercial PSPI, in order to optimize process performance, is usually also added with a coupling agent, a defoamer, a leveling agent and other additives. PSPI is cured into a film after the performance of the film is measured mainly by the structure of the matrix resin and molecular weight, while the photolithography process performance, at the same time, is measured by the structure of the resin and photoinitiators, sensors and other light-sensitive system effects. As with ordinary photoresists, according to the differences in the solubility properties of the exposure and non-exposure areas, PSPI can be divided into positive-working photosensitive polyimide (p-working) and negative-working photosensitive polyimide (n-working). Regardless of p-PSPI or n-PSPI, several photosensitive mechanisms have been developed and commercialized in recent decades.

**Figure 1:**
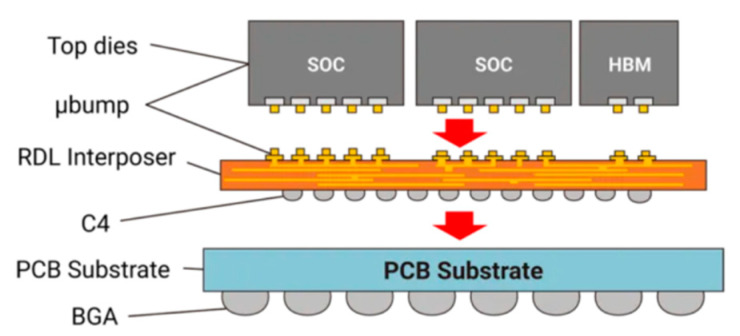
A schematic diagram of the RDL. The image is source from TSMC.

Due to the pursuit of “smaller” chips, in addition to the continuous reduction in transistor line width, the chip packaging process is also rapidly developing and innovating. The key dimensions of the chip process have entered the nanometer era, and the packaging process has evolved to the stage of advanced packaging technology. For example, current widely used redistribution layer (RDL) packaging technology [6] refers to a single layer of integrated circuits coated with a light-sensitive insulating protective layer; this exposure helps to develop the way to define a new wire pattern and then, by using electroplating technology to produce a new metal line, to achieve the purpose of line redistribution. A schematic diagram is shown in Figure 1 [7]. Figure 1 is from the TSMC website, where SoC, HBM, C4, PCB and BGA stand for System on Chip, High Bandwidth Memory, Controlled Collapse Chip Connection, Printed Circuit Board and Ball Grid Array, respectively. The advantages of RDL are threefold: (1) reducing the design cost by replacing a portion of the chip’s internal wiring with the design of the RDL wiring; (2) supporting a larger number of pins; and (3) making the I/O (Input/Output) contact spacing more flexible and the bump area larger, which reduces the stress between the substrate and the components and improves the reliability of the product [8,9].

Considering various elements such as resistivity, heat dissipation and cost, the metal wiring of the RDL is mainly copper, usually plated, with a thickness of 5~10 μm. RDL technology makes the interlayer between the insulating layer and the metal conductor repeatedly and alternately distributed, which has higher requirements for the tightness between the insulating layer and the metal conductor, the resistance to high and low-temperature impacts, the resistance to high-temperature storage, and other properties. PSPI material is widely used as an insulating layer for the RDL, and with the development of Fan-out advanced wafer-level packaging technology, it is now possible to achieve seven-layer polymer and seven-layer metal (7P7M) RDL packages [10]. Since the thermal expansion coefficient of traditional PSPI curing film materials is usually 40~70 ppm, higher than that of copper (15~20 ppm), the difference in the thermal expansion coefficient during thermal curing will produce great stress; so, cracking between the insulating layer and the metal conductor often occurs in aging resistance experiments, and it is the most common failure phenomenon of the interlayer of insulating layer materials. Meng-Hsin et al. manufactured PSPI with a great CTE of 17.9 ppm/K by using the appropriate prescription of a diamine monomer (2,2-bis (3-amino-4-hydroxyphenyl)-hexafluoropropane; APAF) and a dianhydride monomer (4,4′-(4,4′- isopropylidenediphenoxy) bis (phthalic anhydride); BPADA) [11]. Ogura, T et al. developed a negative-type PSPI (n-PSPI) based on PAA (80 wt%) and 2,6-dimethylpiperidine (DNCDP, 20 wt%), which showed high sensitivity of 70 mJ/cm^2^ and a low CTE of 16 ppm/K [12]. Reducing the coefficient of thermal expansion of PSPI and thus reducing the cracking phenomenon is an effective way to realize the further wide application of PSPI [13,14].

In recent years, hyperbranched polymers have attracted attention due to their unique physical and chemical properties and convenient preparation processes [15,16,17,18]. Hyperbranched polymer molecules are a three-dimensional spherical structure with a large number of terminal functional groups on the periphery, low chain entanglement, and peripheral distribution of functional groups, which can give them extremely high functional activity, good solubility and low viscosity. Introducing hyperbranched structures into polyimide macromolecular chains can prepare hyperbranched polyimides with good solubility and mechanical properties [19,20,21].

In this study, an approach for the synthesis of negative PSPI resins with a low coefficient of thermal expansion was devised, and we successfully synthesized this material by designing a three-branched photopolyimide precursor component, as well as by selecting the appropriate light-curing monomers, light-curing initiators, additives and organic solvents. The n-PSPI compositions with a low CTE and low inner stress synthesized in this study are coated, exposed, developed and thermally cured to yield polyimide films with a low coefficient of thermal expansion, low stress, a low dielectric constant, low dielectric loss and low water absorption, with excellent mechanical, chemical-resistant and thermally stable properties, which can be applied to passivation layer films in the semiconductor manufacturing industry and the microelectronic packaging industry. These excellent properties have great industrial scalability and can be applied to the semiconductor manufacturing industry for passivation layer films and the microelectronic packaging industry for insulating layer films, dielectric layer films, the stress buffer protective layer film preparation process, and the multilayer metal wiring interconnection structure of the interlayer dielectric and insulating diaphragm.

## 2. Experimental

### 2.1. Materials

4,4′-oxydiphthalic anhydride (ODPA) and 4,4′-oxydianidine (ODA) were purchased from China-tech (Tianjin, China) Chemical Co., Ltd. 1,3,5-tris(4-aminophenyl) benzene (TAPB) was purchased from Macklin Biochemical Technology (Shanghai, China) Co., Ltd. Dimethylacetamide (DMAc) and N-methyl-2-pyrrolidinone (NMP) were purchased from Concord Technology (Tianjin, China) Co., Ltd. and used as received. 2-Hydroxyethyl methacrylate (HEMA) and thionyl chloride (SOCl_2_) were purchased from Aladdin Bio-Chem Technology (Shanghai, China) Co., Ltd. Other commercially available solvents and reagents were purchased from Inno Chem Sci. Technol. (Shandong, China) Co., Ltd. and used as received.

### 2.2. Synthesis of PAE Resin

**Scheme 1:**
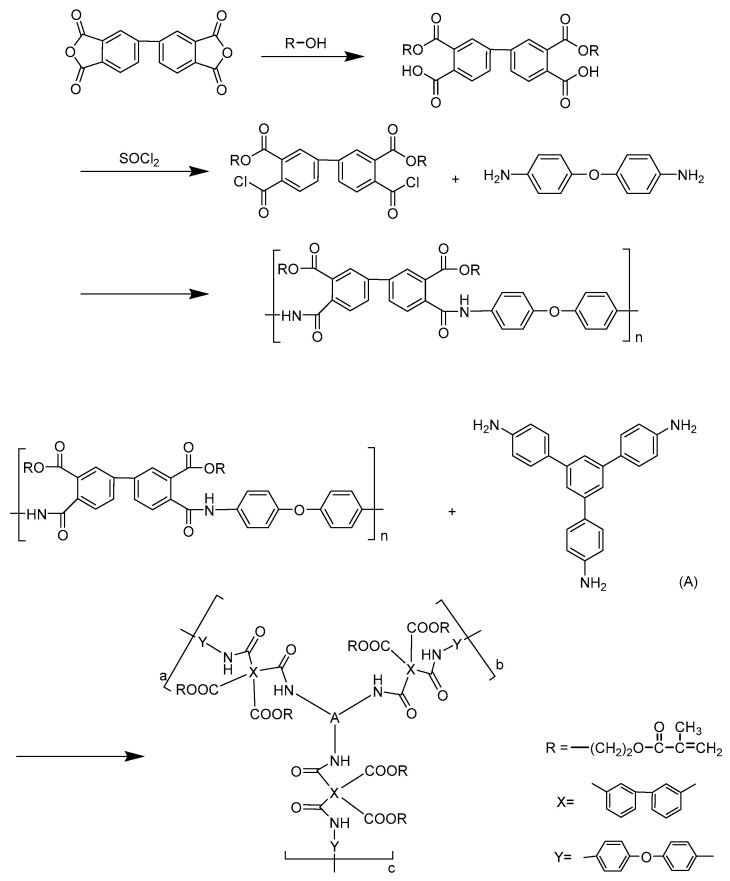
Synthesis of PAE resin.

The synthesis of PAE-1 was prepared as illustrated in Scheme 1.

To a 500 mL three-necked flask equipped with a stirrer, a nitrogen inlet and a thermometer under nitrogen protection, we added 4,4′-Oxydiphthalic anhydride (0.064 mol, 19.85 g) and 2-hydroxyethyl methacrylate (0.128 mol, 16.66 g), which was dissolved with 100 mL of N,N-dimethylacetamide (DMAc). Then, SOCl_2_ (0.128 mol, 15.23 g) was added slowly drop-wise and the reaction was carried out at 0–10 °C for 2 h and at room temperature for 4 h. In addition, 4,4′-Oxydianiline (0.048 mol, 9.61 g) was dissolved in 100 mL of DMAc, and after it was completely dissolved, it was slowly added to the dianhydride solution and reacted overnight. 1,3,5-Tris(4-aminophenyl) benzene (0.01 mol, 3.514 g) was dissolved in 50 mL of DMAc and slowly added drop-wise to a three-necked bottle at room temperature for 24 h during a light-shading reaction to obtain the polyamidoacid resin solution. The above reaction solution was poured into 5 L of deionized water, and the solid was precipitated, filtered and dried under vacuum at a low temperature to obtain the polyamide acid ester resin.

In the yellow light zone, 40 g of the above polyamido acid ester resin, 0.60 g of 1,3-diphenylpropanetrione-2-(O-ethoxycarbonyl) oxime, 0.10 g of N-phenyl diethanolamine, 0.03 g of 2,6-di-tert-butyl-p-methylphenol and 4.0 g of diethyleneglycol dimethacrylate were added sequentially into 80 g of DMAc and stirred for 3 h at room temperature, so that a homogeneous-phase negative photosensitive polyamidate resin composition solution was obtained. Preferably, the resin composition solution may be filtered using filter paper or filter sacs.

### 2.3. Measurements

^1^H NMR spectra were recorded on a Bruker Avance 400 Spectrometer in CDCl_3_ or DMSO-d6. The number average molecular weight (M_n_), the weight average molecular weight (M_w_) and the polydispersity indices (PDIs) were measured by using a gel permeation chromatography (GPC) system (Waterse2695, Milford, MA, USA) using NMP containing 0.02 mol H_3_PO_4_ as the eluent at a flow rate of 0.7 mL/min at 50 °C. Solubility was measured by dissolving 1.0 g of pc-PAE in 9.0 g of organic solvents (10 wt% concentration) with stirring for 24 h at room temperature.

Film thickness was measured by using a Nano Spec II full-automatic film thickness tester (Onto Innovation Inc., Wilmington, MA, USA). Lithographic performance was investigated by using an Olympus MX63 optical microscope (Olympus, Hachimaya, Japan) and a Hitachi s-9380 (Hitachi, Tokyo, Japan) scanning electronic microscope (SEM). Mechanical properties were tested on an Instron-3365 tensile apparatus (Instron, High Wycombe, UK). The glass transition temperature (T_g_) and mechanical properties at high temperatures were analyzed by using a TA Q800 Dynamic Mechanical Analysis (DMA) instrument (Waterse, Milford, USA) under nitrogen. The storage module–temperature curve was obtained at a heating rate of 5 °C/min, and the stress–strain curve at a specific temperature was obtained at a rate of 0.1 N/s. Thermal stability was analyzed by using a TA Q50 Thermogravimetric Analyzer (TGA) instrument (Waterse, Milford, NH, USA) at a heating rate of 20 °C/min under nitrogen. Electrical properties were measured on a Vector network analyzer of Keysight N5227B PNA (Keysight Technologies Inc., Santa Rosa, CA, USA) in 10 GHz.

### 2.4. Photolithography Process

The prepared n-PSPI viscous solution was spin-coated and developed on a CLEAN TRACK™ ACT™ 12 Coater/Developer (Tokyo Electron, Tokyo, Japan), and then exposed on an FPA-5550iZ2 stepper (i-line, Canon, Tokyo, Japan) at 600 mJ/cm^2^. Then, the photopattern on the wafer was developed with cyclopentanone. The developed wafer was cured at 350 °C/2 h in an oxygen-free oven (O_2_ < 100 ppm), and the cured wafer was soaked in a dilute solution of hydrofluoric acid to separate the film from the wafer.

A characteristic photosensitivity curve was obtained by plotting the normalized film thickness against the exposure dose (millijoules per square centimeter). Film thickness on the silicon wafer was measured by using the Veeco Instrument Dektak9 surface profiler (Veeco Instrument Inc., New York, NY, USA). The photopattern was gained by exposing the PI film followed by development and rinsing. After post-baking at 100 °C for 2 h, image-wise exposure through a mask was photographed using the scanning electron microscope.

## 3. Results and Discussion

### 3.1. Synthesis and Characterization of PAE Resins

**Figure 2:**
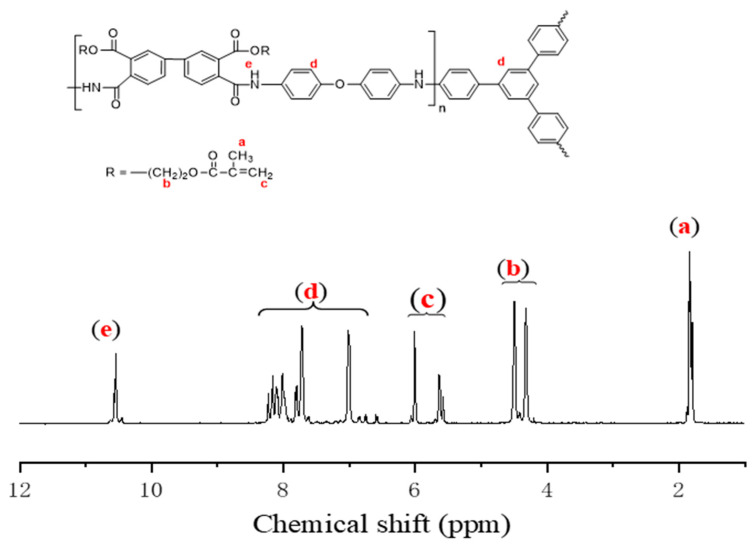
^1^H NMR spectrum of PAE resin.

Figure 2 shows the ^1^H NMR spectra characterizing the chemical structure of PAE. The absorptions at 10.55 ppm (e) and 7.02–8.26 ppm (d) were designated as the protons of amide groups (-CONH-) and of aromatic phenyl groups (Ar-H) in the polymer backbone, respectively. The peaks at 1.87 ppm (a), 4.32–4.50 ppm (b) and 5.62–6.00 ppm (c) were attributed to the protons of -CH_3_, -CH_2_CH_2_O- and =CH_2_ of ethoxylmathacrylate side groups (-CH_2_CH_2_-O-C(O)-C(CH_3_)=CH_2_) in the polymer backbones, indicating that PAE has the expected chemical structure.

**Figure 3:**
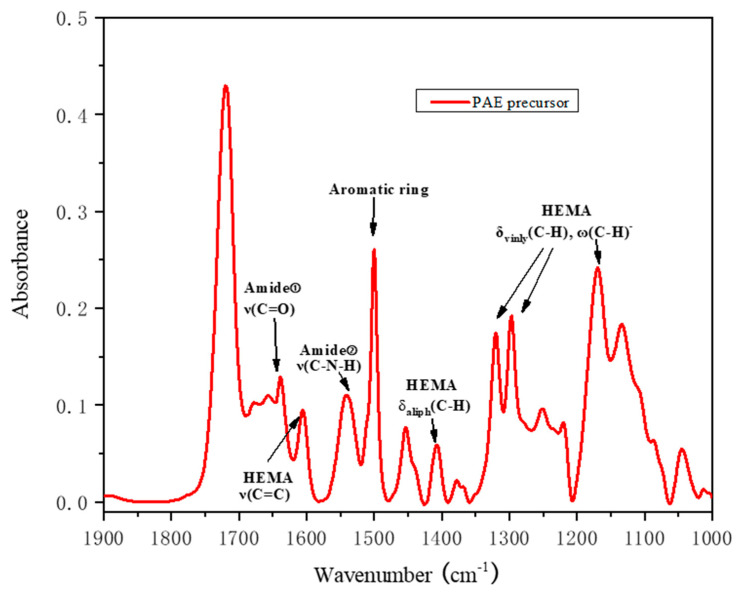
FT−IR spectra of PAE resin.

The FT-IR spectra of the PAE resins were characterized to further demonstrate the chemical molecular structure of the resins. Figure 3 shows that the characteristic absorption of PAE resins at 1720 cm^−1^, 1640 cm^−1^ and 1550 cm^−1^ corresponds to the C=O and C-N telescopic vibration of aromatic amide groups, and the characteristic absorption at 1600–1650 cm^−1^ and 1500 cm^−1^ belongs to an aromatic phenyl group of the C-H telescopic vibration and C-C telescopic vibration. The characteristic absorption of HEMA as a branched chain could also be found at 1604, 1404, 1320, 1290 and 1170 cm^−1^ in the FT-IR spectra, corresponding to the C=C and C-H structures. The FT-IR spectroscopy and ^1^H NMR spectrum findings are consistent in demonstrating that PAE resins have the expected chemical molecular structure.

PAE resins with different molecular weights were synthesized using the same method and named PAE-1, PAE-2, PAE-3, PAE-4 and PAE-5 according to the molecular weight in descending order. The number average molecular weight (M_n_) and mass molecular weight (M_w_) of PAE-1~5 are recorded in Table 1, from which it can be seen that M_n_ was determined in the range of 1.2 × 10^4^–2.0 × 10^4^ g/mol and M_w_ in the range of 2.5 × 10^4^–4.2 × 10^4^ g/mol. The polydispersity index (PDI) of PAE resins can be calculated from the values in Table 1 as 2.1, and the molecular weight distribution of PAE resins can be determined.

### 3.2. Lithographic Performance

The PAE resins, photoinitiators, photocrosslinkers, polymerization inhibitors and adhesion promoters were fully dissolved in a solvent to obtain a viscous solution of n-PSPI, which was spin-coated onto a 6-inch silicon wafer and pre-baked at 110 °C for 4 min to obtain a wet resin film with a thickness of about 10 µm. The pre-baked wet film on the wafer was irradiated with UV light (i line) and developed in cyclopentanone to obtain a three-dimensional PAE film light pattern. The PSPI films were converted into polyimide stereolithographic patterns on the surface of silicon wafers under thermal curing conditions at 350 °C for 2 h. Based on the same formulation, PAE-1~5 resins were configured as PSPI-1~5.

**Figure 4:**
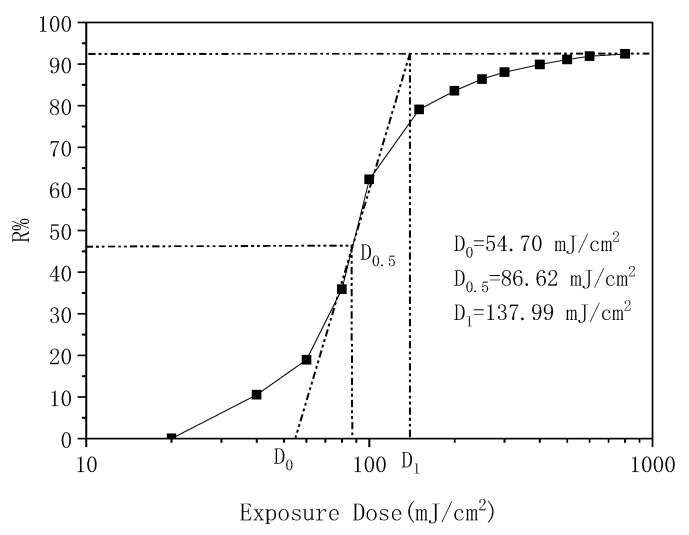
Photosensitivity curves of PSPI-1.

Figure 4 depicts the lithography performance of PSPI-1. The horizontal coordinate is the exposure energy and the vertical coordinate is the film retention rate (R% = post-development film thickness/pre-development film thickness). The photosensitivity curves were determined by normalizing the film thickness at different exposure doses, where D_0_ represents the exposure dose at the beginning of the photochemical reaction, D_0.5_ is the exposure dose at a normalized film thickness of 0.5, which can characterize the sensitivity of the material, and D_1_ represents the exposure dose at the completion of the exposure. γ = [lg(D_1_/D_0_)] − 1 represents the contrast ratio, which reflects the sensitivity of the material, the edge condition of the pattern and the resolution of the material. PSPI-1 prepared with PAE-1 has a sensitivity of 87 mJ/cm^2^ and a contrast of 2.5.

Table 2 compares the lithographic properties of PSPIs prepared with different PAEs. The resolutions of the vias were measured in the range of 8–15 µm in a 5 µm thickness film after being cured at 350 °C/2 h. The sensitivity increased from 87 mJ/cm^2^ (PSPI-1) to 94 mJ/cm^2^ (PSPI-5), and the contrast reduced from 2.5 (PSPI-1) to 1.7 (PSPI-5). It can be seen that the image resolution of PSPI decreases with the increase in the molecular weight of PAE resins, but the overall decrease is not significant, and the decrease in the graphic resolution and contrast is more obvious. Since the proportion of photoinitiators added in the process of configuring different resins into PSPI adhesives is the same, this indicates that the photosensitivity is mainly determined by photoinitiators and less affected by molecular weight when the resins have the same structure. Since the molecular weight directly affects the solubility of the resin in the cyclopentanone developer, it has a greater effect on resolution and contrast. The worse the solubility, the worse the resolution and contrast. It was also observed that the increase in molecular weight resulted in an increase in the optimum development time from 40 s (PSPI-1) to 120 s (PSPI-5). Therefore, by comparing the lithography performance of PSPI-1~5, it is found that PSPI-2 has the best lithography performance and is suitable for industrial scale-up.

**Figure 5:**
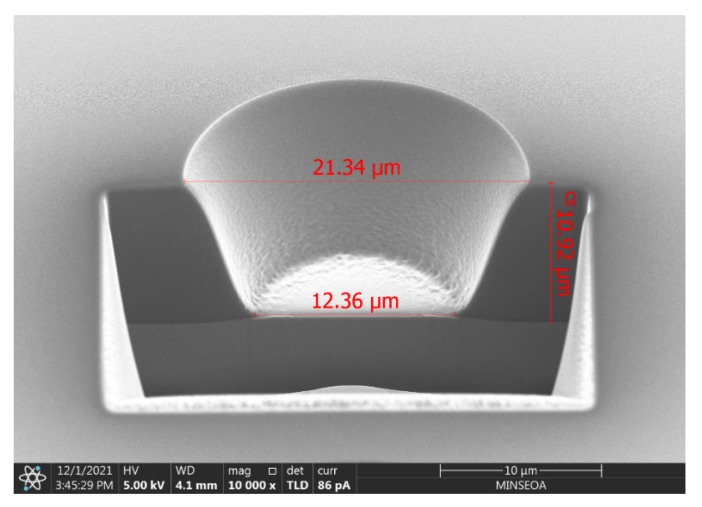
Photopatterning of PSPI-1: via with 15 μm diameter.

Figure 5 shows the photopattern micrograph of PSPI-1. A resolution of 8 µm (via) at an exposure dose of 150 mJ/cm^2^ was obtained in a 5 µm thickness film after being cured at 350 °C for 2 h, as shown in Figure 5. As can be seen in Figure 5, the micropatterns have straight lines and sharp edges. The Focused Ion Beam (FIB) results show that the pattern edges are very sharp, and no obvious pattern collapse, distortion or drift is observed. It can be seen that the prepared PSPI possesses excellent lithographic performance, which is consistent with the results of the photosensitive curve and lithographic specific properties.

### 3.3. Thermal and Dielectric Properties

**Figure 6:**
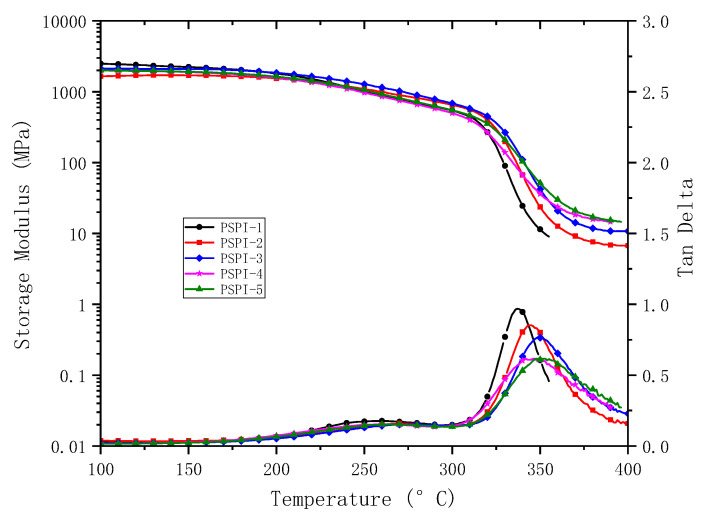
DMA curves of PSPI films thermally cured at 350 °C/2 h in nitrogen.

**Figure 7:**
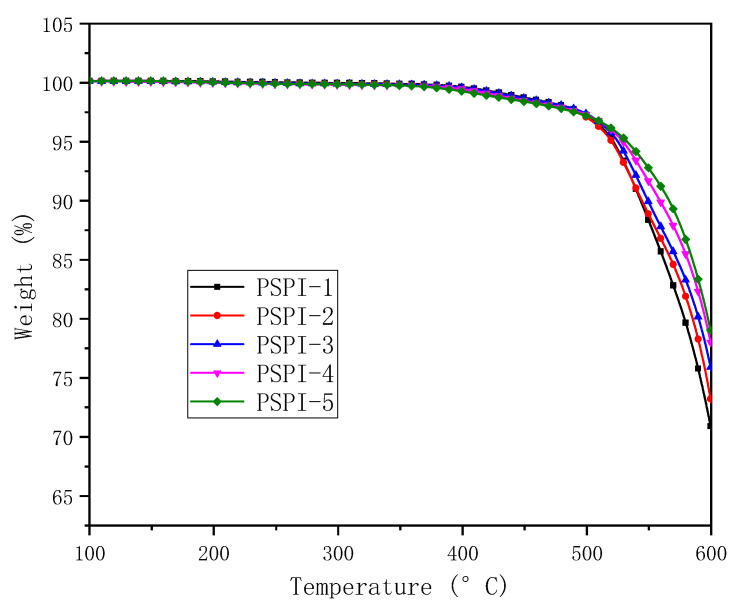
TGA curves of PSPI films thermally cured at 350 °C/2 h in nitrogen.

Through the customized mask and step exposure process, not only can the high-resolution pattern be obtained, but the smooth and uniform mechanical and thermodynamic performance test splines can also be prepared. The thermal properties of PSPI films thermally cured at 350 °C/2 h are summarized in Table 3, which effectively demonstrates the excellent thermomechanical properties of PSPI with a tri-branched structure. Figure 6 compares the DMA curves of PSPI films thermally cured at 350 °C/2 h. The glass transition temperatures (T_g_) were measured in the range of 338–352 °C. All of the cured films did not show significant weight loss before the temperature reached 400 °C in nitrogen (Figure 7). The 5% weight loss temperatures (T_d5_) were measured in the range of 500–529 °C and the 10% weight loss temperatures (T_d10_) in the range of 522–559 °C, indicating that the cured polyimide films exhibit excellent thermal stability.

**Figure 8:**
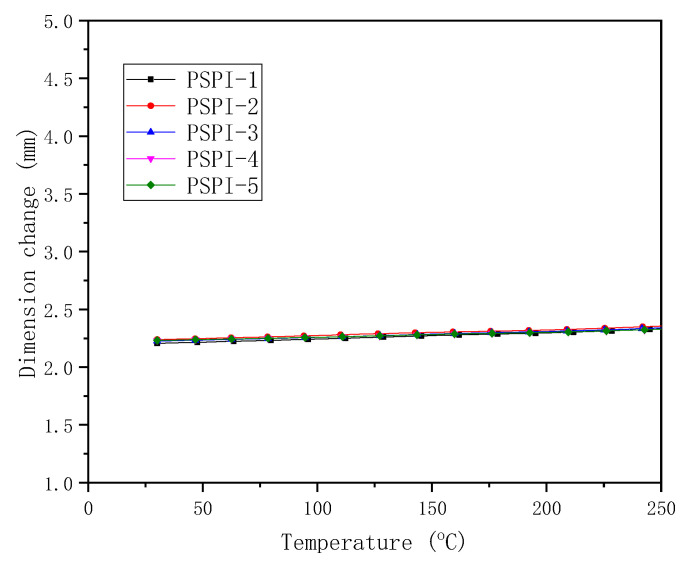
TMA curves of PSPI films thermally cured at 350 °C/2 h in nitrogen.

Figure 8 shows the TMA curves of n-PSPI films thermally cured at 350 °C/2 h. The coefficient of thermal expansion (CTE) values of the films were measured at temperatures ranging from 50 to 150 °C. The CTE values of PSPI-1~5 films were measured to be 46, 41, 38, 29 and 29 ppm/°C, respectively. It can be seen that at temperatures below 150 °C, the polyimide films with tri-branched structures exhibit linear thermal expansion characteristics with low coefficients of thermal expansion.

The dielectric constant (ε′, D_k_) is defined as the ability of an electrolyte to store electrical energy. A higher capacity to store electrical energy implies a higher value of ε′. In addition, the dielectric consumes a portion of the electrical energy to generate heat, called dielectric loss (tan δ, D_f_). Table 4 shows the dielectric constant and dielectric loss of PSPI films thermally cured at 350 °C/2 h. The D_k_ values of each film are between 2.88 and 2.92, and the D_f_ values are between 0.0028 and 0.0033. The fluctuation range of the dielectric loss and dielectric constant of PSPI-1~5 is not large, and it can be determined that the molecular weight of the PSPI films with a tri-branched structure does not have much influence on the electrical properties of the PSPI films, which proves that the PSPI films with this molecular structure have excellent electrical properties.

Table 5 compares the mechanical properties of the thermally cured polyimide films at 350 °C/2 h, including the elongation at breakage (E_b_), tensile modulus (T_M_) and tensile strength (T_s_). The elongation at break of the PSPI samples with a tri-branched structure ranges from 34.6 to 57.6%, the tensile modulus ranges from 3.4 to 3.5 GPa and the tensile strength ranges from 131.5 to 182.0 MPa, which proves that n-PSPI resins with different molecular weights and a tri-branched structure have good mechanical properties, and that all of the parameters tend to rise with the increase in molecular weight. All of the parameters show an upward trend with the increase in molecular weight.

### 3.4. Practical Applications in RDL

**Figure 9:**
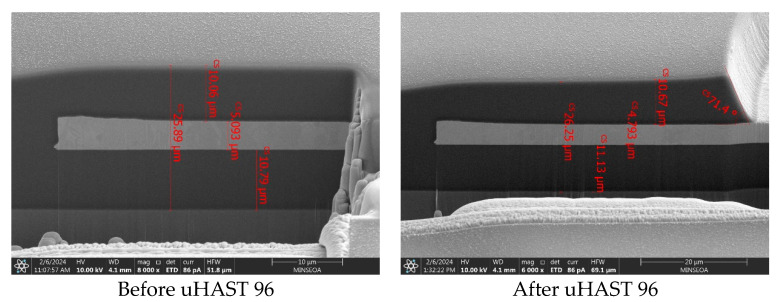

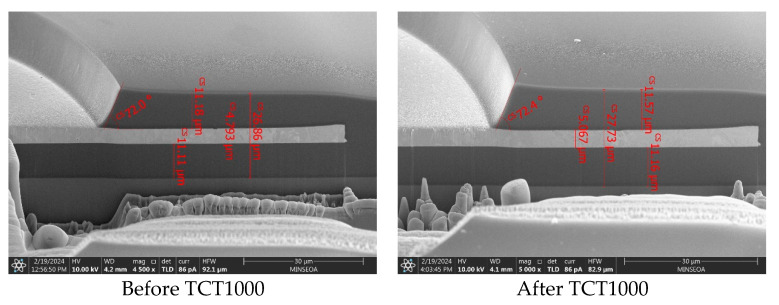
Reliability test results of PSPI-2 in 2P2M-RDL packaging (uHAST 96 and TCT1000).

In the field of electronic chip packaging, collar packaging materials and chip reliability verification are particularly important. Because the failure of packaging materials can lead to chip failure, which in turn affects the performance and lifetime of electronic products, the reliability verification of chips is very strict, and the existing conventional reliability test schemes are shown in Table 6 [22,23]. After RDL package validation and reliability testing of the PSPI-2 samples according to the reliability test schemes listed above, FIB-SEM was used to observe the bonding of the package material and the metal conductor in the cross-section. Figure 9 shows the SEM photographs of the PSPI-2 samples before and after different reliability test validations (uHAST 96, TCT1000) after the completion of the 2P2M (two polymer layers, two metal layers) RDL process. From the figure, it can be seen that there is not much change before and after the reliability test, which proves that there is no cracking or other failure between the PSPI-2 cured thin film material and the metal conductor, which thus proves that the PSPI-2 samples have good compatibility with the copper substrate and have a practical application in the field of RDL encapsulation.

## 4. Conclusions

PAE-1~5 resins with different molecular weights were synthesized and the corresponding PSPI-1~5 negative electrode materials were prepared using the same formulations. The molecular weights and ^1^H NMR and FT-IR characterization of the PAE resins confirmed that they had the expected chemical structures. After thermal curing at 350 °C/2 h in nitrogen, the PSPI films exhibited excellent mechanical, thermal and electrical properties. The lithographic properties of the PSPI films with different molecular weights, such as resolution and photosensitivity, were characterized, and it can be seen that the lithographic properties of PSPI films with a tri-branched structure are not much affected by the molecular weight, which proves that the structure can effectively avoid the influence of molecular weight in relation to its lithographic properties. The dielectric constant and dielectric loss of PSPI-1~5 were also tested, which further confirmed that the molecular weight of the photosensitive polyimide with a tri-branched structure has little effect on the electrical properties. The micrographs of the n-PSPI samples before and after reliability testing and validation were characterized, confirming that they did not show any cracking or other failure phenomena between the cured thin film material and the metal conductor of PSPI-2, proving that the PSPI-2 samples have good compatibility with the copper substrate and are of practical application value in the field of RDL packaging. This PSPI film was also shown to have a low coefficient of thermal expansion (<20 ppm), low residual stress (<20 MPa) and a high resolution close to that of copper. The negative PSPIs have been validated for use in 12-inch silicon wafers, primarily as interlayer dielectric insulation for multilayer metal interconnect circuits. Also, this negative PSPI resin composition is particularly well suited for large (8 to 12 inch) thick film packages due to its low coefficient of thermal expansion and low stress.

## Figures and Tables

**Table 1:** Molecular weights of different PAEs.

PAEs	Molecular Weight		
	M_n_ (×10^4^ g/mol)	M_w_ (×10^4^ g/mol)	PDI
PAE-1	1.2	2.5	2.1
PAE-2	1.4	2.9	2.1
PAE-3	1.6	3.2	2.1
PAE-4	1.8	3.8	2.1
PAE-5	2.0	4.2	2.1

**Table 2:** Lithographic properties of PSPIs.

PSPIs	Resolution (µm)	Sensitivity (mJ/cm^2^)	Contrast
PSPI-1	8	87	2.5
PSPI-2	8	90	2.3
PSPI-3	10	93	2.0
PSPI-4	12	93	1.9
PSPI-5	15	94	1.7

**Table 3:** Thermal properties of PSPI films thermally cured at 350 °C/2 h in nitrogen.

PSPIs	CTE (TMA, ppm/°C)	T_g_ (DMA, °C)	Decomposition Temperature (°C)	
			T_d5_	T_d10_
PSPI-1	46	338	500	522
PSPI-2	41	343	520	544
PSPI-3	38	350	505	529
PSPI-4	29	344	529	559
PSPI-5	29	352	520	554

**Table 4:** Dielectric properties of PSPI films thermally cured at 350 °C/2 h in nitrogen.

PSPIs	D_k_, ε′ (10 GHz)	D_f_, tanδ (10 GHz)
PSPI-1	2.89	0.0033
PSPI-2	2.92	0.0031
PSPI-3	2.90	0.0032
PSPI-4	2.88	0.0028
PSPI-5	2.90	0.0031

**Table 5:** Mechanical properties of n-PSPI films thermally cured at 350 °C/2 h.

PSPIs	*E*_b_ (%)	*T*_M_ (GPa)	*T*_s_ (MPa)
PSPI-1	34.6	3.4	131.5
PSPI-2	42.4	3.4	153.7
PSPI-3	46.5	3.5	162.7
PSPI-4	46.5	3.5	169.0
PSPI-5	57.6	3.5	182.0

**Table 6:** Reliability verification test conditions for packaging.

Item.	Conditions	Standards
Precon condition	125 °C, 24 h	JESD22-A113C
MSL3	30 °C, 60% RH, 192 h	JESD22-A113
uHAST96	RT~260 °C, 3 cycle	JESD22-A110B
TCT1000	130 °C, 85% RH, 2 atm, 96 h	JESD22-A104D
MR3X+UHAST96	Condition B, −55~125 °C Stay 5 min, 20 min/cycle, 500 cycle	JESD22-A110B
MR5X+TCC200	150 °C, 500 h	JESD22-A104D

## Data Availability

The data presented in this study are available on request from the corresponding author.
